# Recent Developments in the Speciation and Determination of Mercury Using Various Analytical Techniques

**DOI:** 10.1155/2015/372459

**Published:** 2015-07-05

**Authors:** Lakshmi Narayana Suvarapu, Sung-Ok Baek

**Affiliations:** Department of Environmental Engineering, Yeungnam University, Gyeongsan-si 712 749, Republic of Korea

## Abstract

This paper reviews the speciation and determination of mercury by various analytical techniques such as atomic absorption spectrometry, voltammetry, inductively coupled plasma techniques, spectrophotometry, spectrofluorometry, high performance liquid chromatography, and gas chromatography. Approximately 126 research papers on the speciation and determination of mercury by various analytical techniques published in international journals since 2013 are reviewed.

## 1. Introduction

**Figure 1:**
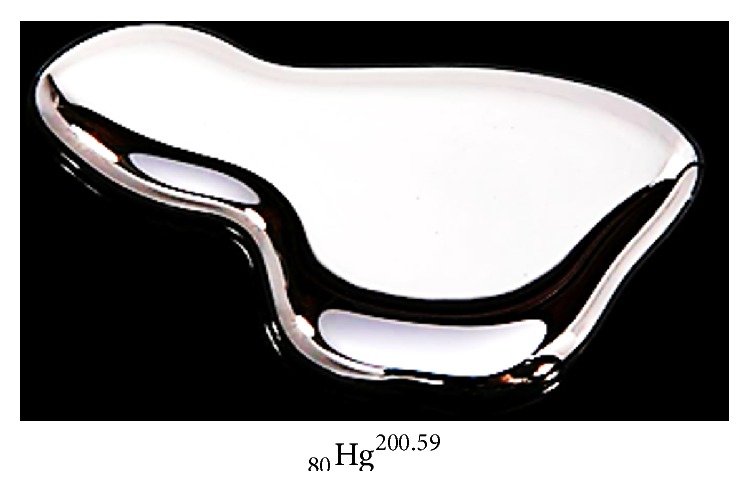
Elemental mercury.

Mercury, which is also known as quick silver, is only the metal (Figure 1) in the modern periodic table that exists in liquid form at room temperature. The sources of mercury in the environment include the natural processes, such as breakdown of minerals in rocks and volcanic activities. The anthropogenic sources are not limited to mining and the burning of fossil fuels. Regarding the toxicity of mercury and its different species, methylmercury poisoning affects the nervous system of humans and damages the brain and kidneys [1]. Most of the mercury emitted into the environment is converted to methylmercury, which spreads to the food chain due to the bioaccumulation nature of methylmercury [2]. Owing to the toxicity nature and bioaccumulation nature of mercury, most studies in this area have focused on the determination of mercury and its species in various environmental and biological samples.

Marumoto and Imai [3] reported the determination of dissolved gaseous mercury in the seawater of Minamata Bay of Japan. This study also estimated the exchange of mercury across the air-sea interface. Panichev and Panicheva [4] reported the determination of the total mercury content in fish and sea products by thermal decomposition atomic absorption spectrometry. Fernández-Martínez et al. [5] evaluated different digestion systems for the determination of mercury with CV-AFS (cold-vapor atomic fluorescence spectrometer) in seaweeds. Pinedo-Hernández et al. [6] examined the speciation and bioavailability of mercury in sediments that had been impacted by gold mining in Colombia.

This paper presented the recent developments in this topic after a previous review published in 2013 [2]. The present study reviews the recent developments in the speciation and determination studies of mercury reported and published since 2013. For this purpose, approximately 136 research papers published were reviewed. All the analytical parameters such as limit of detection, linearity range, and interference study reported by the reviewed papers are presented in Tables 1–4 [7–133]. This extensive collection of literature and the analytical parameters of the reviewed papers established the recent developments in the determination and speciation studies of mercury using a range of analytical techniques.

## 2. Discussion

The toxicity and bioaccumulation nature of mercury has prompted extensive studies to determine the concentrations of mercury species in different environmental and biological samples. This paper reviewed a large number of studies on the determination and speciation of toxic metals including mercury. The reviews regarding the determination of mercury published since 2013 are discussed hereunder.

Suvarapu et al. [2] reviewed research papers published between 2010 and 2011 regarding the speciation and determination of mercury using a variety of analytical techniques. They concluded that most researchers prefer cold-vapor atomic absorption spectrometry (CV-AAS) and atomic absorption spectrofluorometry (CV-AFS) for the speciation and determination studies of mercury in various environmental samples. Suvarapu et al. [134] also reviewed research papers published in 2012 regarding the determination of mercury in various environmental samples. El-Shahawi and Al-Saidi [135] reviewed the dispersive liquid-liquid microextraction (DLLME) method for the speciation and determination of metal ions including mercury. This review concluded that the method of DLLME has the advantages of simplicity, speed, and low cost for the determination of metal ions using various analytical techniques. Ferreira et al. [136] reviewed the use of reflux systems for the sample preparation in the determination of elements, such as arsenic, antimony, cadmium, lead, and mercury. This study concluded that the use of the reflux systems is very rare in the determination of elements, such as Hg. Gao et al. [137] reviewed the application of chemical vapor generation method for the determination of metal ions, such as mercury and cadmium with ICP-MS. Sańchez et al. [138] reviewed the determination of trace elements including mercury present in petroleum products using ICP techniques. This study concluded that the electrothermal vaporization and laser ablation methods were promising for the analysis of petroleum for trace elements. Martín-Yerga et al. [139] reviewed the determination of mercury using electrochemical methods. This study discussed the advantages and disadvantages of the use of different electrodes in the determination of mercury. Chang et al. [140] reviewed the detection of heavy metals, such as cadmium, lead, and mercury in water samples using graphene based sensors. This study concluded that it is a very challenging task to detect heavy metals in water in real time due to the interference of large chemical and biological species in water. Yu and Wang [141] reviewed the determination of metal ions including mercury by atomic spectrometry by applying flow-based sample pretreatment methods. They concluded that the ICP-AES, AAS, AFS, and ICP-MS are the major detection techniques for trace metal analysis. Yin et al. [142] reviewed the speciation analysis of mercury, arsenic, and selenium using a range of analytical techniques. Gao and Huang [143] reviewed the determination of mercury(II) ions by voltammetry and concluded that stripping voltammetry is still an active field of research regarding the determination of mercury. Duarte et al. [144] reviewed disposable sensors and electrochemical sensors for the environmental monitoring of Pb, Cd, and Hg. They recommended the recycling of materials used in sensors for future studies. Recently, Ferreira et al. [145] reviewed the analytical strategies of sample preparation for the determination of mercury in food matrices.

In recent days, few research papers were published about the determination and analysis of mercury species in various environmental and biological samples and some of them are discussed hereunder. Lima et al. [146] reported an efficient method for the determination of mercury in inorganic fertilizers by using CV-AAS combined with microwave-induced plasma spectrometry. Pelcová et al. [147] reported the simultaneous determination of mercury species by LC-AFS with a low detection limit of 13–38 ng L^−1^. Chen et al. [148] reported a colorimetric method for the determination of mercury ions based on gold nanoparticles and thiocyanuric acid. Fernández et al. [149] reported gold nanostructured screen-printed carbon electrodes for the determination of mercury using dispersive liquid-liquid microextraction. Fernández-Martínez et al. [5] evaluated the different digestion systems for determination of mercury in seaweeds using CV-AFS. Silva et al. [150] determined the trace amounts of mercury in alcohol vinegar samples collected from Salvador, Bahia of Brazil. Jarujamrus et al. [151] reported a colorimetric method using unmodified silver nanoparticles for the determination of mercury in water samples. A highly selective method for the determination of mercury using a glassy carbon electrode modified with nano-TiO_2_ and multiwalled carbon nanotubes in river and industrial wastewater was reported by Mao et al. [152].

As mentioned in our previous review [2], spectrometric techniques are used widely by many researchers for the determination of mercury over the world. Regarding the determination of mercury with various analytical instruments in the papers reviewed, more than 55% of the researchers used spectrometric instruments, such as atomic absorption spectrometry (AAS), inductively coupled plasma techniques (ICP-OES, AES, and MS), and atomic fluorescence spectrometer (AFS) (Table 1). ICP-MS technique has an advantage of low detection limits and wide range of linearity in the determination of mercury [153]. Around 20% of the researchers chose the spectrophotometer and spectrofluorometer (Table 2) for the determination and speciation of mercury. Approximately 10% of researchers in the papers reviewed used electrochemical instruments for the determination and speciation studies of mercury (Table 3). Only a few authors chose the HPLC, GC, and other techniques (Table 4) but they coupled these instruments with AAS or other instruments. Regarding the analysis of the environmental biological samples for mercury and its species, most researchers analyzed various water samples (drinking, seawater, wastewater, river, and lake waters) followed by food samples (mostly fish), human hair, and ambient air. Only a few authors determined the concentration of mercury in ambient air and atmospheric particulate matter [26, 48, 52, 66, 119, 126]. Various measurement techniques that can be available for the determination of mercury species in ambient air were reviewed by Pandey et al. [154]. This study also concluded that most of the researchers preferred CV-AAS and CV-AFS technique for the measurement of different mercury species in ambient air. In comparison of methods, acid digestion and thermal method, for the analysis of mercury in ambient air acid digestion, is better than thermal method. By the thermal methods the values can be obtained 30% lower than the acid digestion method [155].

In the analysis of mercury species in various environmental samples, selectivity and range of linearity of the method also play a major role due to the presence of multielements in the real samples. Based on the present study, most of the spectrophotometric, spectrofluorometric, and electroanalytical methods were discussed regarding the interfering ion studies and linearity range of the method. These studies will give a clear picture about the determination of mercury species in presence of other ions which validates the methods.

Regarding the merits of the different methods for speciation and analysis of mercury, the usage of nonchromatographic methods has an advantage in terms of speed of analysis, inexpensiveness, and convenience to find the mercury in various environmental samples. But for the complete speciation studies of mercury in biological and environmental samples chromatographic methods are useful [156]. The validity of analytical methods can be enhanced with the analysis of the certified reference materials along with the real samples. In recent years, the researchers mostly preferred GC coupled with AFS or ICP-MS for the determination and speciation of mercury in natural waters [157]. In electroanalytical methods, the validity of the methods depends on various factors such as type of electrode, preconcentration, and supporting materials [139] and these methods are cost-effective, selective, and sensitive [143].

## 3. Conclusions

The present study revealed the recent developments in the determination and speciation studies of mercury by a range of analytical techniques. Our previous study [2] also described the challenges in the methodology for mercury determination. This review showed that most researchers focused on the determination of Hg(II) rather than speciation studies. On the other hand, the speciation studies [23, 24, 29, 36, 37, 44, 47, 50, 54, 58, 68, 69, 76, 118] accurately revealed the toxicity of mercury rather than the total mercury or single species determinations. In the papers reviewed, most researchers were aware of the interfering ions in the determination of mercury and its different forms. In the analytical method, a study of interfering ions is very important because it can predict the selectivity of the method. In future studies, it will be important to focus on speciation studies of mercury rather than a determination of the total mercury.

## Figures and Tables

**Table 1:** Analytical parameters of reviewed research papers about the speciation and determination of mercury by spectrometric instruments (AAS, ICP-OES, AES, MS, and AFS).

S. number	Analyte	Analytical instrument used for the detection	Method	Limit of detection (LOD)^#^	Linearity range	Analyzed samples	Interference study	Supporting media	Reference
1	Total Hg	CV-AAS and ICP-AES	Microwave acid digestion	4.83 × 10^−10^ M	—	Fish samples	Cadmium and lead also analyzed along with mercury	—	[7]

2	Hg(II)	CV-AAS	Preconcentration	1.79 × 10^−10^ M	—	Water and human hair	Recovery of Hg^2+^ is in the range of 95.6–104.9% in presence of Cu^2+^, Co^2+^, Zn^2+^, Ni^2+^, Cd^2+^, Mn^2+^, Ba^2+^, Pb^2+^, Fe^3+^, Cr^3+^, Al^3+^, Ag^+^, K^+^, Na^+^, NH_4_^+^, Mg^2+^, and Ca^2+^ ions, from 750 to 2500-fold	Dithizone	[8]

3	Total Hg	CV-AAS	Ultrasound extraction	6.98 × 10^−11^ M	—	Alcohol vinegar	—	—	[9]

4	Total Hg	CV-AAS	SPE^1^	4.98 × 10^−11^ M		Rice, canned fish, and tea leaves	The tolerance limit for Na^+^, K^+^, Mg^2+^, and Ca^2+^ is 4000-fold, for Ba^2+^ and Zn^2+^ is 40-fold, for Fe^3+^, Cr^3+^, Co^2+^, and Ni^2+^ is 10-fold, and for Al^3+^ is 200-fold compared to Hg^2+^	Fe_3_O_4_ nanoparticles	[10]

5	Hg(II)	CV-AAS	SPE	9.97 × 10^−12^ M	Up to 500 *μ*g L^−1^	Water samples	As, Al, Fe, Mo, and Sb are depressed the Hg signal	Carbon nanotubes	[11]

6	Total Hg	CV-AAS	Acid digestion	3.6 × 10^−9^ M	—	Marine fish	—	—	[12]

7	Total Hg	CV-AAS	Wet digestion	3.0 × 10^−9^ M	—	Green tiger shrimp	Arsenic also determined along with mercury	—	[13]

8	Total Hg	CV-AAS	Alkaline fusion digestion	0.06 ng g^−1^	0.006–4000 ng g^−1^	Phosphate rock	—	—	[14]

9	THg	AAS	Acid digestion	4.98 × 10^−12^ M	—	Fish muscle tissues	Cadmium and lead also detected along with mercury	—	[15]

10	Hg(II)	CV-AAS	SPE	1.19 × 10^−11^ M	0.01–2.30 *μ*g L^−1^	Water samples	Fe^3+^, Cu^2+^, Zn^2+^, Cd^2+^, Co^2+^, and Mn^2+^ are not interfered up to 5 mg L^−1^ and NH_4_^+^ and Tl^3+^ are not interfered up to 1 mg L^−1^	Polymer supported ionic liquid	[16]

11	Hg(II)	CV-AAS	SPE	9.97 × 10^−11^ M	0.07–2.00 *μ*g L^−1^	Water samples	Tolerable amount of major metals is limited up to 50 *μ*g L^−1^	Polytetrafluoroethylene	[17]

12	Total Hg	CV-AAS	Digestion	3.98 × 10^−10^ M	2.5–10.0 *μ*g L^−1^	Biological samples	—	Cold finger	[18]

13	Total Hg	CV-AAS	Combustion	2.99 × 10^−13^ M	—	Water and fish	Arsenic and selenium also determined along with mercury	—	[19]

14	Total Hg	AAS	Amalgamation	0.2 ng/g for hair and 0.02 ng/g for blood	—	Hair and blood samples	Arsenic and selenium also determined along with mercury	—	[20]

15	Total Hg	CV-AAS (total Hg) and GC-ICPMS (MeHg)	Cold-vapor reduction with NaBH_4_	5.98 × 10^−11^ M (total Hg) and 2.3 × 10^−9^ M (MeHg)	—	Blood of birds	Selenium also determined along with mercury and methylmercury	—	[21]

16	Total Hg and MeHg	CV-AAS (total Hg) and CV-AFS (MeHg)	Digestion	0.03–0.1 ng/g	—	Fish, vegetables, and mushrooms	Selenium and cadmium also determined with mercury species	—	[22]

17	Hg speciation	CV-AAS (total Hg) and CV-AFS (MeHg)	Acid digestion	—		Water samples	—	—	[23]

18	Hg speciation	CV-AAS	LLME^2^	1.49 × 10^−10^ M (Hg^2+^) and 1.8 × 10^−9^ M (MeHg)	0.5–100 ng mL^−1^	Water samples and CRMs	The recovery of Hg^2+^ in presence of foreign ions is 95–105 and for MeHg is 96–106%	—	[24]

19	Total Hg	GF-AAS	Acid mineralization	6.97 × 10^−11^ M	—	Fish muscle samples	—	Copper nitrate	[25]

20	GEM	AAS				Ambient air	—	—	[26]

21	Total Hg	CV-AAS	Acid digestion	0.0006 *μ*g g^−1^	—	Freshwater fish samples	—	Stannous chloride	[27]

22	Total Hg	AAS	Combustion	0.01 ng	—	Soil samples	Interference of various heavy metals was overcome by using sample pretreatment	—	[28]

23	Hg speciation	AAS (THg) and ICP-MS-HPLC (MeHg)	Hydride generation	5.33 × 10^−14^ M	20 *μ*g L^−1^	Fish samples	Cd, Pb, As, and Sn also measured along with Hg	—	[29]

24	THg	HG-AAS	Hydride generation	98.4% (accuracy)	—	Irrigation water wells	Along with mercury Pb, Cd, and Al Cr also measured	—	[30]

25	THg	CV-AAS and AAS	Thermal decomposition and amalgamation	1.34 × 10^−9^ M (TD-amalgamation AAS) and 3.14 × 10^−9^ M (CV-AAS),	—	Soil samples	—	—	[31]

26	Total Hg speciation	HV-AAS and HPLC-CV-AFS	Extraction	—	—	Aqueous solutions and fish tissue	—	Multiwalled carbon nanotubes	[32]

27	Total Hg	CV-AAS (DMA)	Microwave oven digestion	—	—	Canned fish	Selenium and tin also measured along with mercury	—	[33]

28	THg	AMA (AAS)	AAS principles and without digestion process	—	—	Fish red muscle and white muscle	—	—	[34]

29	THg	AES	LIBS and SIBS	2 × 10^−3^ M (LIBS) and 9.97 × 10^−5^ M (SIBS)		Soil samples	At 534.074 nm has less spectral interference	—	[35]

30	Hg speciation	CV-AFS	Extraction	1.0 (total Hg) and 0.01 MeHg ng g^−1^	—	Sea water and sediments	—	—	[36]

31	Hg speciation	CV-AFS	Extraction	0.01 × 10^−12^ M (Hg^0^) and 0.002 × 10^−12^ M (DM Hg)	—	Sea waters	—	—	[37]

32	Total Hg	CV-AFS	Microwave assisted digestion	3.98 × 10^−13^ M	—	Nuts	Interference of fat in nuts is removed by treatment with chloroform and methanol	—	[38]

33	Hg(II)	AFS	Fluorescence optical sensor	9.57 × 10^−12^ M	2.27 × 10^−11^–1.13 × 10^−3^ M	Human hair, urine, and well water samples	Most of the alkali, alkaline, and transition metal ions did not interfere in the determination of Hg^2+^	N-(2-Hydroxy phenyl)-N-(2-mercapto phenyl)-o-phthalylidene	[39]

34	GEM	CV-AFS	Gold amalgamation	0.0002 ng	—	Total suspended particulates	—	QFF (quartz fiber filters)	[40]

35	MeHg	AAS and CV-AFS	Acid digestion	0.005 *μ*g/g	—	Water, soil, sediments, and foodstuffs	—	—	[41]

36	Total Hg	CV-AFS	Microwave assisted digestion	0.5 ng g^−1^	—	Sediments	—	Sequential injection system	[42]

37	Total Hg	CV-AFS	Acid digestion	0.48 ng g^−1^	—	Rice	Interference of other metal ions is eliminated by acid wash and kept storage of samples for 24 h	Multisyringe flow injection analysis	[43]

38	Hg speciation	HPLC-AFS	UV-induced atomization	1.9 × 10^−9^ (Hg^2+^), 1.9 × 10^−9^ (MeHg), and 2.0 × 10^−9^ (EtHg) M		CRMs	—	—	[44]

39	Hg(II)	UV-AFS	SPE	1.49 × 10^−13^–3.98 × 10^−13^ M	1–5000 ng L^−1^	Natural waters	10 mg L^−1^ of Fe^2+^, Fe^3+^, Cu^2+^, Pb^2+^, and As^3+^ and 10 g L^−1^ of Na^+^, K^+^, and Ca^2+^ did not interfere in the determination of 100 ng L^−1^ of Hg^2+^	Sodium diethyldithiocarbamate	[45]

40	Hg(II)	AFS	Micro-SPE	5.98 × 10^−11^ M	Up to 5 *μ*g L^−1^	Water samples	—	Mesofluidic platform	[46]

41	Hg speciation	EX-AFS		0.5 ng g^−1^ (total Hg)	—	Waste calcines	—	—	[47]

42	Hg speciation	CV-AFS	Extraction	~0.5 pg	—	Atmospheric air	—	PTFE filter papers	[48]

43	MeHg	GC-AFS	SPE	12 ng g^−1^	Up to 1.5 ng mL^−1^	Biological samples	—	—	[49]

44	Hg speciation	HPLC-AFS	Liquid-liquid microextraction	1.54 × 10^−10^ (Hg^2+^), 7.42 × 10^−11^ (MeHg), 1.045 × 10^−10^ (EtHg), and 3.31 × 10^−10^ M (PhHg)	0.0–20 *μ*g L^−1^	Environmental waters	No interference from other metal ions	1-Octyl-3-meth-l imidazolium hexafluorophosphate	[50]

45	MeHg	CV-AFS	Extraction	0.515 ng g^−1^	—	Petroleum	—	TMAH^3^, KOH/CH_3_OH, HCl, and acidic CuSO_4_/KBr	[51]

46	GEM	CV-AFS	—	—	—	Ambient air	—	—	[52]

47	Hg(II)	AFS	Fluorescence	0.07 × 10^−6^ M	0.1–4.5 *μ*M	Aqueous solutions	Longer excitation and emission wavelength could shield the interference	Fe_3_O_4_ magnetic nanoparticles	[53]

48	Hg speciation	CV-AFS	Thermal decomposition	—	—	Fish liver	Method validity is tested with CRM	—	[54]

49	THg	AFS	—	<4.98 × 10^−12^ M	—	Snow	—	K_2_Cr_2_O_7_/SnCl_2_	[55]

50	Atmospheric Hg	CV-AFS	Extraction	—	—	Particulate matter	—	—	[56]

51	THg	CV-AFS	Flow injection mercury system	—	—	Herbal products	—	Protease papain	[57]

52	Hg speciation	LC-UV-CV-AFS	Microwave digestion	4.98 × 10^−12^ (total Hg), 1.39 × 10^−12^ (MeHg), and 1.99 × 10^−12^ (Hg^2+^) M	—	Sea food	Simultaneously determined both Hg(II) and MeHg	—	[58]

53	MeHg and total Hg	CV-AAS	Digestion	0.088 (MeHg) and 0.005 (total Hg) *μ*g g^−1^	—	Hair and milk of mothers	—	—	[59]

54	Hg(II)	ICP-MS	Microfluidic	3.49 × 10^−10^ M	0.2–4.0 *μ*g L^−1^	Aqueous samples	The recovery of Hg^2+^ in the presence of 100 *μ*g L^−1^ of Ca^2+^, Cd^2+^, Co^2+^, Cr^3+^, Cu^2+^, K^+^, Mg^2+^, Na^+^, Ni^2+^, Pb^2+^, and Zn^2+^ is in the range of 97.5–101.7%	Gold nanoparticles	[60]

55	Total Hg	ICP-MS	Acid digestion	0.053–0.01 *μ*g g^−1^	—	Pharmaceutical ingredients	Low residual carbon content in digests is desirable to minimize some interference	—	[61]

56	Hg(II)	ICP-MS	Adsorption	—	—	Wastewaters	—	Multiwalled carbon nanotubes	[62]

57	Hg(II)	ICP-OES	Extraction	1.49 × 10^−11^ M	—	Fish samples	Selective in presence of Na^+^, K^+^, Cs^+^, Ca^2+^, Mg^2+^, Zn^2+^, Fe^2+^, Cu^2+^, Co^2+^, Ni^2+^, Mn^2+^, Cd^2+^, and Pb^2+^ into 1 mg L^−1^ solutions of Hg(II) in pH 8	Ion imprinted polymer	[63]

58	Total Hg	CV-ICP-MS	Microwave digestion	3 ng g^−1^	—	Plants and soil	—	—	[64]

59	Total Hg	ICP-MS	Microwave assisted digestion	—	—	Rice	—	—	[65]

60	GEM	CV-ICP-MS	Thermal analysis	20 × 10^−15^ g	—	Atmospheric particulates	—	—	[66]

61	Hg(II) and MeHg	HPLC-ICPMS	HF-LPME^4^	5.48 × 10^−10^ (Hg^2+^) and 1 × 10^−9^ (MeHg) M	Up to 50 *μ*g L^−1^	Tap, river, and estuarine waters	Simultaneously selenium also determined along with mercury	—	[67]

62	Hg speciation	ICP-MS	Ion exchange chromatography	9.47 × 10^−11^ (Hg^2+^), 1.25 × 10−10 (MeHg), 1.35 × 10^−10^ (EtHg), and 7.92 × 10^−10^ (PhHg) M	0.1–100 *μ*g L^−1^ (all Hg species)	Sea water and marine fish	—	L-Cysteine or thiourea	[68]

63	Hg speciation	GC-ICP-MS	Preconcentration	27 (Hg^2+^) and 12 ng g^−1^ (MeHg)	—	Human hair	—	—	[69]

64	Total Hg	MC-ICPMS	Isotope ratio analysis	0.1–0.2 disintegrations per minute	—	Sediment core	Mercury and mercury isotope compositions are determined	—	[70]

65	Hg(II) and MeHg	CVG-ICP-MS	Extraction	1.7 (Hg(II)) and 2.3 ng g^−1^ (MeHg)	—	Fish samples	—	—	[71]

66	MeHg, Hg(II), and EtHg	HPLC-CV-ICPMS	Extraction and separation	5.98 × 10^−11^ (Hg(II)), 2.17 × 10^−11^ (EtHg), and 1.8 × 10^−8^ (MeHg) M	—	Plasma/serum samples	—	—	[72]

67	Total Hg	ICP-MS	Microwave assisted digestion	—	—	Freshwater fish samples	—	—	[73]

68	Total Hg	ICP-MS	Isotope dilution and UV-photochemical vapor generation	0.5 pg g^−1^	—	Biological tissues	Polyatomic interference is not detectable	Formic acid	[74]

69	Total Hg	ICP-MS	Calcination-isotope dilution	2 × 10^−15^ M	—	*Diploria *specimens	No isobaric interference was found	—	[75]

70	Hg speciation	ICP-MS	Anion exchange chromatographic separation	3.98 × 10^−11^ (Hg^2+^), 1.11 × 10^−10^ (MeHg), 1.26 × 10^−10^ (EtHg), and 1.22 × 10^−10^ (PhHg) M	—	Fish samples	—	3-Mercapto-1-propanesulfonate	[76]

71	Total Hg	ICP-MS	Ultrasonic slurry sampling electrothermal vaporization	0.2 ng g^−1^	—	Herbal samples	As, Cd, and Pb also determined along with Hg	8-Hydroxyquinoline	[77]

72	Total Hg	ICP-MS	Electrothermal vaporization	5.98 × 10^−11^ M	—	Water associated with crude oil production	By preconcentration of analyte interference is avoided	—	[78]

73	THg	ICP-MS	Isotope dilution equation	4.98 × 10^−11^ M for THg	0.0005–1.321 mg/kg for MeHg	Arctic cod	—	—	[79]

^#^For the conversion of limit of detection values into moles per liter (M) the atomic weight of Hg is taken as 200.59 g, MeHg as 215.59 g, EtHg as 229.59 g, and PhHg as 277.59 g.

^1^Solid-phase extraction; ^2^LLME: liquid-liquid microextraction; ^3^TMAH: tetramethylammonium hydroxide; ^4^HF-LPME: hallow fiber liquid phase microextraction.

Analytical instruments: CV-AAS: cloud vapor atomic absorption spectrometer; HG-AAS: hydride generation AAS; GF-AAS: graphite furnace AAS; ICP-OES: inductively coupled plasma optical emission spectrometer; ICP-MS: ICP-mass spectrometer; ICP-AES: ICP-atomic emission spectrometer; HPLC: high performance liquid chromatography; AFS: atomic fluorescence spectrometer; AMA: automatic mercury analyzer; DMA: direct mercury analyzer.

**Table 2:** Analytical parameters of reviewed research papers about the speciation and determination of mercury by spectrophotometer and spectrofluorometer.

S. number	Analyte	Analytical instrument used for the detection	Method	Limit of detection (LOD)^#^	Linearity range	Analyzed samples	Interference study	Supporting media	Reference
1	Hg(II)	Fluorescence spectrophotometer	Fluorescence	4.0 × 10^−9^ M	6.0–450 nM	Water samples	10-fold of Pb^2+^, Cu^2+^, and Ag^+^ shows <7% influence on the determination of Hg^2+^ compared to reported ones	CdTe quantum dots	[80]

2	Hg(II)	Spectrophotometer	Colorimetric	23 × 10^−9^ M	0.00–0.31 *μ*M	River water	Selective in presence of Ag^+^, Cd^2+^, Cu^2+^, Co^2+^, Ni^2+^, and Pb^2+^	Carbon nanodots	[81]

3	Hg(II)	Spectrophotometer	Colorimetric	2.6 × 10^−9^ M	0.001–1 *μ*M	Water samples	Selective in presence of 20 *μ*M of Al^3+^, Ca^2+^, Co^2+^, Cu^2+^, Cd^2+^, Fe^3+^, Mn^2+^, Ni^2+^, Pb^2+^, and Zn^2+^	Gold nanoparticles	[82]

4	Hg(II)	Spectrophotometer	Colorimetric	—	0.83–8.6 *μ*g mL^−1^	Water samples	The tolerance limit of Cu^2+^, V^5+^, Ag^+^, Pd^2+^, Pt^4+^, Au^3+^, Fe^2+^, Ni^2+^, Cd^2+^, Pb^2+^, and Cr^6+^ is in the range of 0.11–041 *μ*g mL^−1^in the determination of 1.91 *μ*g mL^−1^ of Hg^2+^	5-Methylthiophene-2-carboxaldehyde ethylenediamine	[83]

5	Hg(II)	Spectrofluorometer	Fluorescence	1.73 × 10^−9^ M	2.0 nM–60 *μ*M	—	Interference of major cations studied	ONPCRs^1^	[84]

6	Hg(II)	Spectrophotometer	Colorimetric	50 × 10^−9^ M^2^	0–1000 nM	Water samplers	Selective in presence of Ni^2+^, Co^2+^, Ca^2+^, Cu^2+^, Na^+^, K^+^, As^3+^, Mg^2+^, Cd^2+^, and Fe^2+^	Silver nanoparticles	[85]

7	Hg(II)	UV-Vis spectrophotometer	Colorimetric	1.35 × 10^−6^ M	—	Drinking water	Cd^2+^, Pb^2+^, Fe^3+^, and Ba^2+^ do not interfere in the determination of Hg^2+^ but Mg^2+^, Ca^2+^, and Mn^2+^ interfere slightly	Gold nanoparticles	[86]

8	Hg(II)	Spectrofluorometer and UV-spectrometer	Colorimetric and fluorescent sensor	2.7 × 10^−8^ M	0–1.0 × 10^−6^ M	Water samples and living cells	The fluorescent signal for Hg(II) is not influenced by the major metal ions including Fe(III), Cu(II), and Al(III)	2,4-Dichloroquinazoline	[87]

9	Hg(II)	Spectrophotometer	Colorimetric	5.3 × 10^−13^ M	1.0 × 10^−12^–8.6 × 10^−4^ M	Water samples and SRM	Selective in presence of Mn^2+^, Fe^2+^, Fe^3+^, Ni^2+^, Co^2+^, Cd^2+^, and Pb^2+^	Chromoionophore V	[88]

10	Hg(II)	Spectrofluorometer	Fluorescent and colorimetric	1.0 × 10^−9^ M	—	Spiked water samples	Na^+^, Mg^2+^, K^+^, Cr^3+^, Mn^2+^, Co^2+^, Ni^2+^, Fe^3+^, Cu^2+^, Zn^2+^, Ag^+^, Cd^2+^, and Pb^2+^ did not interfere	Rhodamine B	[89]

11	Hg(II)	Spectrofluorometer	Fluorescence	14.2 × 10^−9^ M	0–5 × 10^−7^ M	Aqueous solutions	Cd^2+^, Cu^2+^, and Ag^+^ do not interfere	Thioether-appended dipeptide	[90]

12	Hg(II)	Spectrofluorometer	Fluorescence	0.5 × 10^−9^ M	0.0005–0.01 *μ*M	Lake water samples	Zn^2+^, Pb^2+^, Ni^2+^, Ca^2+^, Mg^2+^, Cu^2+^, Co^2+^, Cd^2+^, Fe^3+^, and Mn^2+^ did not interfere	Carbon nanotubes	[91]

13	Hg(II)	Spectrofluorometer	Fluorescent	1.74–3.83 × 10^−6^ M	—	Living cells	Minor interference from Ag^+^, Ca^2+^, Cd^2+^, Co^2+^, Cu^2+^, Fe^2+^, Fe^3+^, K^+^, Mg^2+^, Mn^2+^, Na^+^, Ni^2+^, Pb^2+^, Rb^+^, and Zn^2+^	Pyrene	[92]

14	Hg(II)	Spectrophotometer	Colorimetric	0.4 × 10^−6^ M	0.1–4.2 *μ*g mL^−1^	Water, biological, plant leaves, and soil samples	Tolerance limit of the Cd^2+^, Zn^2+^, Ce^3+^, Ce^4+^, In^3+^, Cr^3+^, La^3+^, Yb^3+^, and Eu^3+^ is 300 *μ*g mL^−1^ and the tolerance limit of the Co^2+^, Cu^2+^, Fe^3+^, Ti^4+^, Pb^2+^, Ni^2+^, and Ag^+^ is 100 *μ*g mL^−1^ and at Hg(II) is 2.0 *μ*g mL^−1^	2,4,7-Triamino-6-phenylpteridine	[93]

15	Hg(II)	Spectrofluorophotometer	Fluorescent	1.0 × 10^−7^ M	2.0 × 10^−7^–3.0 × 10^−5^ M	Water samples	Selective in presence of Na^+^, K^+^, NH_4_^+^, Ba^2+^, Zn^2+^, Cd^2+^, Mg^2+^, Ca^2+^, and Ni^2+^	Conjugated polymer multilayer films	[94]

16	Hg(II)	Spectrophotometer	TGFRET^3^	0.49–0.87 × 10^−9^ M	1.0 × 10^−9^–1.0 × 10^−8^ M	Water samples	Selective in presence of Mn^2+^, Ba^2+^, Ni^2+^, Cu^2+^, Ca^2+^, Cr^2+^, Co^2+^, Cd^2+^, Mg^2+^, Zn^2+^, Al^3+^, Fe^3+^, and Pb^2+^	Gold nanoparticles	[95]

17	Hg(II)	Spectrofluorometer	Fluorescent	1 × 10^−9^ M	0.01–0.12 *μ*M	Water samples	Selective in presence of Zn^2+^, Pb^2+^, Ni^2+^, Co^2+^, Ca^2+^, Cu^2+^, Mg^2+^, Cd^2+^, Fe^3+^, and Mn^2+^	Carbon nanodots	[96]

18	Hg(II)	Spectrofluorometer	Fluorescent	0.012 × 10^−6^ M	0-1 *μ*M	Tap and river water samples	Selective in presence of Ag^+^, Pb^2+^, Na^+^, K^+^, Cr^3+^, Cd^2+^, Ba^2+^, Zn^2+^, Mg^2+^, Cu^2+^, Ni^2+^, Ca^2+^, Al^3+^, and Fe^3+^	Rhodamine	[97]

19	Hg(II)	Spectrofluorometer	Fluorescence	2.24 × 10^−9^ M	5.0–100 nM	Drinking water	20-fold of Ca^2+^, Mg^2+^, Zn^2+^, Cr^3+^, Pb^2+^, Cr^6+^, Mn^2+^, Cd^2+^, Fe^3+^, Al^3+^, and Ni^2+^, 10-fold of Fe^2+^, and Co^2+^, 5-fold of Cu^2+^, and the same concentration of Ag^+^ caused almost no interference	Gold nanoparticles	[98]

20	Hg(II)	Spectrophotometer	Optical chemical sensor	0.18 × 10^−12^ M	7.2 × 10^−13^–4.7 × 10^−4^ M	Tap water, river water, and canned tuna fish	Interference of Cu(II) eliminated with the addition of L-histidine as a masking agent	Synthesized ionophore	[99]

21	Hg(II)	UV-Vis spectrophotometer	Colorimetric sensor	5.0 × 10^−6^ M (visual), 1.0 × 10^−7^ M (UV-Vis)	—	Aqueous solutions	Mg^2+^, Ca^2+^, Zn^2+^, Cu^2+^, Cr^3+^, Fe^3+^, Pb^2+^, Ni^2+^, Co^2+^, and Ag^+^ did not interfere	Dimethyl sulphoxide	[100]

22	Hg(II)	Fluorescence spectrophotometer	Fluorescence probe	16 × 10^−9^ M	0.02–1.0 *μ*M	Aqueous solutions	Selective in the determination of Hg^2+^ over other metal ions such as Fe^3+^, Ca^2+^, Mg^2+^, Mn^2+^, Cr^3+^, Ni^2+^, Cu^2+^, Co^2+^, and Pb^2+^	Gold nanoparticles	[101]

23	Hg(II)	—	Colorimetric	1.2 × 10^−9^ M	2–30 nM	Water samples	Na^+^ (2 mM), K^+^ (2 mM), Fe^3+^, Zn^2+^ and Mg^2+^ (0.1 mM), Ni^2+^, Co^2+^, Cd^2+^, Pb^2+^ and Cu^2+^ (50 *μ*M), and Ag^+^ (3.5 *μ*M) did not interfere with the detection of Hg^2+^ (25 nM) in the mentioned amounts	Rhodamine B thiolactone	[102]

^#^For the conversion of limit of detection values into moles per liter (M) the atomic weight of Hg is taken as 200.59 g, MeHg as 215.59** **g, EtHg as 229.59** **g, and PhHg as 277.59** **g.

^1^ONPCRs: oxygen-doped nitrogen-rich photoluminescent polymer carbon nanoribbons; ^2^Limit of quantification; ^3^TGFRET: time-gated fluorescence resonance energy transfer.

**Table 3:** Analytical parameters of reviewed research papers about the speciation and determination of mercury by electrochemical instruments.

S. number	Analyte	Analytical instrument used for the detection	Method	Limit of detection (LOD)^#^	Linearity range	Analyzed samples	Interference study	Supporting media	Reference
1	Hg(II)	DP-ASV	Electrochemical	4.99 × 10^−8^ M	—	Ambient water, tap, and wastewaters	Palladium-natural phosphate-carbon paste electrode enhances the selectivity for Hg^2+^	Natural phosphate electrodes	[103]

2	Hg(II)	SW-ASV	Electrochemical	0.04 × 10^−6^ M	0.2–10.0 *μ*M	Foodstuffs	Simultaneously both Cd^2+^ and Hg^2+^ are determined and 1,000-fold for K^+^, Na^+^, Li^+^, NH_4_^+^, Ca^2+^, Mg^2+^, Pb^2+^, Zn^2+^, Cr^3+^, Fe^2+^, Co^2+^, and Al^3+^ did not interfere	Carbon paste electrode	[104]

3	Hg(II)	Differential pulse voltammeter	Electrochemical	4.48 × 10^−10^ M	0.2–10 *μ*g L^−1^	Spiked fish and plant samples	Cu(II), Mg(II), As(III), and Cr(II) were possible interferers	4,4′-Bipyridine-silver polymer	[105]

4	Hg(II)	Cyclic voltammeter	Electrochemical	0.8 × 10^−14^ M	10^−14^–10^−7^ M	—	Cu^2+^, Pb^2+^, Ni^2+^, Zn^2+^, Cr3^+^, Co^3+^, As^5+^, Fe^2+^, and Fe^3+^ did not interfere	Gold atomic cluster-chitosan	[106]

5	Hg(II)	Voltammeter (cyclic and differential pulse)	Biosensor	3.93 × 10^−12^ M	0.005–0.034 mM	Water samples	The working potential controlled to minimize the interference of other metal ions in test medium	PANI and PANI-co-PDTDA polymer films	[107]

6	Hg(II)	ASV	Electrochemical	4.98 × 10^−9^ M	4–160 ppb	Aquatic solutions	—	Glassy carbon electrode	[108]

7	Hg(II)	SW-ASV	Electrochemical	9.2 × 10^−5^ M	0.1–150.0 nM	Soil, gasoline, fish, tap, and wastewaters	400-fold mass ratio of Cu^2+^, Mn^2+^, Zn^2+^, Cr^3+^, Cr^6+^, Fe^3+^, Fe^2+^, Ni^2+^, and Co^2+^ did not interfere in the simultaneous determination of Cd^2+^, Pb^2+^, and Hg^2+^	Triphenyl phosphine	[109]

8	Hg(II)	Potentiometer	Electrochemical	9.77 × 10^−6^ M (PME)^1^ 7.76 × 10^−7^ M (CGE)^1^	1.0 × 10^−1^–5.0 × 10^−6^ M (PME) 1.0 × 10^−1^–5.0 × 10^−7^ M (CGE)	Water samples	Ag^+^ has small interference in the determination of Hg^2+^	1,3-Alternate thiacalix[4]crown	[110]

9	Hg(II)	Potentiometer	Electrochemical	1.0 × 10^−8^ M	5.0 × 10^−8^–1.0 × 10^−2^ M	—	The selectivity coefficient of the other ions is ranging from 2.9 to 4.9	PVC membrane	[111]

10	Hg(II)	DPSV	Electrochemical	0.05 × 10^−12^ M	1–500 nM	Water samples	Pb^2+^, Th^3+^, Cu^2+^, Cd^2+^, Ni^2+^, and Al^3+^ did not interfere	Gold nanoparticles	[112]

11	Hg(II)	SW-ASV	Ultrasonic extraction	—	—	Indoor dust samples	—	Gold nanoparticles	[113]

12	Hg(II)	Cyclic voltammeter	Electrochemical	1.9 × 10^−9^ M	40–170 *μ*g L^−1^	Wastewaters	—	Biotinyl Somatostatin-14 peptide	[114]

13	Hg(II)	Potentiometer	Electrochemical	3 × 10^−6^ M	5 × 10^−6^–1 × 10^−2^ M	Contaminated water	Na^+^, K^+^, Mg^2+^, Ca^2+^, Zn^2+^, Cu^2+^, Cr^3+^, Fe^3+^, and Pb^2+^ did not interfere in the determination of Hg^2+^	Dithizone and di-n-butyl phthalate	[115]

14	Hg(II)	DP-ASV	Electrochemical	0.483 × 10^−6^ M	300–700 ng mL^−1^	—	No interference of Cd, Ni, Zn, and Cu in 50-, 25-, 100-, and 5-fold in excess, respectively	Nanocellulosic fibers	[116]

15	Hg(II)	—	Electrochemical	0.5 × 10^−9^ M	1.0 nM–1.0 *μ*M		Zn^2+^, Mg^2+^, Ca^2+^, Pb^2+^, Cd^2+^, Mn^2+^, Cu^2+^, Ni^2+^, and Fe^3+^ did not interfere	G-quadruplex–hemin (G4–hemin)	[117]

^#^For the conversion of limit of detection values into moles per liter (M) the atomic weight of Hg is taken as 200.59 g, MeHg as 215.59 g, EtHg as 229.59 g, and PhHg as 277.59 g.

^1^PME: polymeric membrane electrode and CGE: coated graphite electrode.

Analytical instruments: DP-ASV: differential pulse anodic stripping voltammeter; SW-ASV: square wave anodic stripping voltammeter.

**Table 4:** Analytical parameters of reviewed research papers about the speciation and determination of mercury by miscellaneous techniques.

S. number	Analyte	Analytical instrument used for the detection	Method	Limit of detection (LOD)^#^	Linearity range	Analyzed samples	Interference study	Supporting media	Reference
1	Speciation	Continuous mercury analyzer	Thermal desorption	—	—	Solid samples (fly ash)	—	—	[118]

2	GEM	Portable mercury analyzer	—	—	—	Atmosphere	—	—	[119]

3	Hg(II)	SERS^1^	—	2.24 × 10^−12^ M	0.001–0.5 ng mL^−1^	Drinking water samples	Selective in presence of Zn^2+^, Mg^2+^, Fe^3+^, Cu^2+^, Pb^2+^, and Mn^2+^	Gold nanoparticles	[120]

4	Hg(II)	HPLC	SPE	1.99 × 10^−10^–4.48 × 10^−9^ M	2.7–300 *μ*g L^−1^	Water samples	Simultaneously Ni^2+^, Co^2+^, and Hg^2+^ are determined	Carbon nanotubes	[121]

5	Hg(II)	SERS	—	0.1 × 10^−9^ M	0.1–1000 nM	Groundwater	Ag^+^ was also determined along with Hg^2+^ and K^+^, Cu^2+^, Ag^+^, Cr^3+^, Fe^3+^, NH_4_^+^, Ca^2+^, Co^2+^, Cd^2+^, and Zn^2+^ did not interfere	Oligonucleotide-functionalized magnetic silica sphere	[122]

6	Total Hg	AMA	Acid digestion	—	—	Eggs and blood of *Eretmochelys imbricata *	Along with mercury Cd, Cu, Zn, and Pb are also determined	—	[123]

7	Hg(II)	Luminescence spectrometer	Fluorescence	3.0–9.0 × 10^−9^ M	0.05–1.0 *μ*M	Water samples	Fairly selective in presence of Ag^+^, Fe^3+^, Zn^2+^, Ca^2+^, Mn^2+^, Mg^2+^, Co^2+^, Pb^2+^, Ni^2+^, Cd^2+^, and Cu^2+^	Silver nanoclusters	[124]

8	Hg(II)	X-ray fluorescence spectrometer	Preconcentration	4.98 × 10^−12^ M	Upto 20 mg L^−1^	Drinking water	—	Activated carbon	[125]

9	Total Hg	DMA		0.14 ng	—	Particulate matter	—	GF/C filters	[126]

10	MeHg and EtHg	HPLC	Chemiluminescence	0.16 ng g^−1^	0.5–20 ng Hg	Soil and sediment samples	Back extraction and another chemical process make the method selective for MeHg and EtHg	Emetine dithiocarbamate	[127]

11	Total Hg	CV-CCPM-OES^2^	Microwave digestion	2.39 × 10^−11^ M	0.27–55 mg kg^−1^	Soil samples	—	—	[128]

12	Hg(II)	Chemodosimeter	Fluorescence	1.71 × 10^−9^ M	1.0 × 10^−7^–1.0 × 10^−6^ M	Blood serum of mice	—	Rhodamine	[129]

13	Hg(0)	XRF	Acid digestion	9.97 × 10^−8^ M	—	Soils from industrial complex	—	—	[130]

14	Total Hg	DMA	Combustion	0.12 ng	0.5–5 ng	Soil and leaf samples	—	—	[131]

15	MeHg and Hg(II)	GC-MS	Matrix solid-phase dispersion	0.06 (MeHg) and 0.12 (Hg(II)) *μ*g/g	—	Tuna fish, angel shark, and guitarfish	—	—	[132]

16	GEM	—	Concentration-weighted trajectory model	—	—	Particulate matter	—	QFF	[133]

^#^For the conversion of limit of detection values into moles per liter (M) the atomic weight of Hg is taken as 200.59 g, MeHg as 215.59 g, EtHg as 229.59 g, and PhHg as 277.59 g.

^1^SERS: surface enhanced Raman scattering; ^2^CV-CCPM-OES: cold-vapor capacitively coupled plasma microtorch fluorescence spectrometry.

Analytical instruments: HPLC: high performance liquid chromatography; AMA: automatic mercury analyzer; DMA: direct mercury analyzer; XRF: X-ray fluorescence.
